# Irisin/BDNF signaling in the muscle-brain axis and circadian system: A review

**DOI:** 10.7555/JBR.37.20230133

**Published:** 2023-12-28

**Authors:** Alexey N. Inyushkin, Vitalii S. Poletaev, Elena M. Inyushkina, Igor S. Kalberdin, Andrey A. Inyushkin

**Affiliations:** Department of Human & Animal Physiology, Samara National Research University, Samara 443011, Russia

**Keywords:** irisin, brain-derived neurotrophic factor, peroxisome proliferator-activated receptor γ coactivator 1α, circadian rhythm, circadian system, muscle-brain axis

## Abstract

In mammals, the timing of physiological, biochemical and behavioral processes over a 24-h period is controlled by circadian rhythms. To entrain the master clock located in the suprachiasmatic nucleus of the hypothalamus to a precise 24-h rhythm, environmental zeitgebers are used by the circadian system. This is done primarily by signals from the retina *via* the retinohypothalamic tract, but other cues like exercise, feeding, temperature, anxiety, and social events have also been shown to act as non-photic zeitgebers. The recently identified myokine irisin is proposed to serve as an entraining non-photic signal of exercise. Irisin is a product of cleavage and modification from its precursor membrane fibronectin type Ⅲ domain-containing protein 5 (FNDC5) in response to exercise. Apart from well-known peripheral effects, such as inducing the "browning" of white adipocytes, irisin can penetrate the blood-brain barrier and display the effects on the brain. Experimental data suggest that FNDC5/irisin mediates the positive effects of physical activity on brain functions. In several brain areas, irisin induces the production of brain-derived neurotrophic factor (BDNF). In the master clock, a significant role in gating photic stimuli in the retinohypothalamic synapse for BDNF is suggested. However, the brain receptor for irisin remains unknown. In the current review, the interactions of physical activity and the irisin/BDNF axis with the circadian system are reconceptualized.

## Introduction

Skeletal muscle is considered an organ expressing hundreds of secreted peptides, called "myokines", in response to contraction. Myokines, muscle-derived polypeptides with autocrine, paracrine and endocrine signaling, can affect the functions of muscle tissue itself as well as many other tissues and organs (*e.g.*, fatty tissues, livers, bones, and brains)^[1]^. A decade ago, irisin, a contraction-regulated myokine with peripheral and central activities, was discovered^[2]^. It was found that during exercise, irisin was secreted into the circulation and drove the transformation of white adipocytes into "brite" cells (brown-like adipocytes), thus brite cells were the product of white adipocytes that turn "beige" under the influence of irisin^[3]^. Moreover, irisin is cleaved from the membrane fibronectin type Ⅲ domain-containing protein 5 (FNDC5), which is in turn produced under the control of the peroxisome proliferator-activated receptor γ coactivator 1α (PGC-1α)^[2]^. The production of irisin in the skeletal muscle of humans and mice was increased by exercise; specifically, in humans, a two-fold increase in plasma irisin levels was observed after 10 weeks of regular and supervised endurance exercise; while in mice, plasma irisin concentrations were significantly increased by 65% after three weeks of free wheel running^[2]^. These data reveal the involvement of irisin in adaptation to physical activity. However, investigators are particularly interested in the effects of irisin on brain functions. Studies showed that irisin induced the expression of brain-derived neurotrophic factor (BDNF) in mouse hippocampal neurons, which was initiated by both the elevated concentrations of peripheral irisin and its forced production in the primary culture of hippocampal cells^[4–5]^.

In mammals, the daily timing of physiological, endocrine and behavioral processes, such as body temperature, sleep/wake cycles, feeding schedule, and hormone production is controlled by circadian rhythms^[6]^. The circadian system is formed of a circuit of molecular clock mechanisms found in nearly all body cell types. Notably, over 40% of mammal genes possess circadian rhythm in mRNA expression^[7–8]^. The circadian system has a hierarchical organization. The master clock located in the suprachiasmatic nucleus (SCN) above the optic chiasm, near the third ventricle, coordinates the downward synchronization of cellular clocks residing in organs and tissues^[9]^. The intrinsic period of the molecular clocks usually slightly differs from 24 h^[10]^. Therefore, these molecular clocks need to be entrained by environmental zeitgebers. The ability for entrainment (synchronizing the clock phase with the timing of environmental cues), enabling a better adaptation of the circadian clock, is of great importance. There is a main and photic mechanism of entrainment, which utilizes light as a zeitgeber, as well as the complex of non-photic mechanisms of entrainment, where various environmental stimuli (*e.g.*, food, exercise, temperature, and stress) act as the zeitgebers^[11]^.

In the current review, the link between physical activity and the irisin/BDNF axis with the circadian system is reconceptualized.

## Endogenous circadian cellular clock machinery

**Figure 1:**
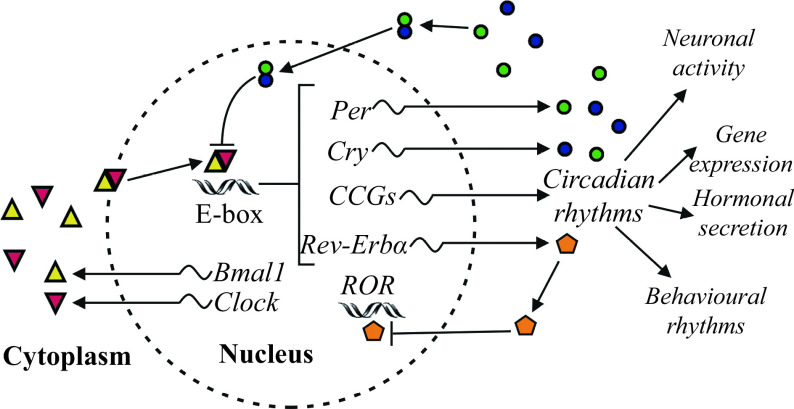
A simplified molecular model of the mammalian circadian oscillator.

The circadian system of mammals is believed to be a result of evolutionary adaptation to environmental cycles resulting from the rotation of our planet about its axis, providing an important advantage by the capability to anticipate future regular daily events in the environment. Circadian rhythm is generated by endogenous circadian cellular core clock machinery relying on interlocked transcriptional-translational feedback loops (TTFLs). A simplified model of the TTFLs that constitute the mammalian circadian clock is shown in ***Fig. 1***.

Autonomous clocks reside in virtually every cell. The core clock machinery contains interacting TTFLs that complete the entire activity cycle for one day. In mice, the central players in TTFLs are brain and muscle ARNT-like protein 1 (BMAL1) as well as circadian locomotor output cycles kaput (CLOCK), which interact together to constitute the central complex BMAL1:CLOCK in the cytoplasm. This complex, positively regulating the circadian cycle, translocates into the nucleus and binds to the E-box in the promotor of three Period genes (*Per1*, *Per2*, and *Per3*) and two Cryptochrome genes (*Cry1* and *Cry2*) in mice^[12]^. These genes represent the negative arm of TTFLs driving the transcription of circadian repressors PER and CRY. After their translation in the cytoplasm, the repressors dimerize and move into the nucleus. Upon nuclear accumulation, heterodimeric protein complexes PER:CRY repress the transcriptional activity of BMAL1:CLOCK, representing a negative feedback completed on a diurnal time. At night, casein kinase 1 (CK1) and adenosine monophosphate kinase (AMPK) phosphorylate the proteins PER and CRY, then the proteins are inactivated and finally degraded by the proteasome. Thus, the repressive effect of PER and CRY is turning off, allowing for the onset of a following circadian cycle. Additionally, in mice, *Bmal1* and *Clock* transcription is repressed by nuclear receptor subfamily 1 group D member 1 (NR1D1, also called Rev-erbα), representing the second negative feedback responsible for the repression of genes *Bmal1* and *Clock*, and the elements of Rev-erbα are supposed to be responsible for the metabolism and circadian rhythm integration^[12]^. In the SCN, core clock genes are rhythmically expressed under the control of the calcium/cAMP response element binding protein (CREB), which is activated by membrane depolarization, intracellular Ca^2+^ and cAMP. Being an active transcription factor, the activated CREB was found to bind to calcium/cAMP regulatory elements of the *Per1* and *Per2* genes to stimulate their transcription in mice^[13]^.

Ancillary to driving circadian oscillations, TTFLs control the expression of thousands of clock-controlled genes (CCGs) responsible for rhythmic circadian fluctuations of physiological, biochemical and behavioral parameters. Molecular clocks optimize the timing of molecular and cellular events, providing conditions for the precise temporal regulation of metabolism. Notably, it has been found that the muscular circadian transcriptome is characterized by highly enriched transcripts of the most important metabolic genes, and the molecular clock temporally regulates the activity of metabolic rate-limiting enzymes in the skeletal muscles^[14]^. Moreover, the muscular clock has been found to synchronize with the master SCN clock, but motor activity and feeding may also be zeitgebers, allowing the skeletal muscle to anticipate regular circadian cues in the environment and to respond adequately to metabolic challenges^[15]^.

As a regulator of irisin expression, PGC-1α also serves as an essential element of the circadian clock. It was reported that PGC-1α could modify the microenvironment of chromatin to an active state, and induced the expression of *Bmal1* and *Clock* genes and subsequent translation in mice^[12]^. Furthermore, studies found that PGC-1α was expressed rhythmically, and THE deacetylation of PGC-1α by sirtuin 1 (SIRT1) contributed to tuning its activity to follow a circadian rhythm^[16–17]^. Besides, the production of PGC-1α was known to be stimulated by factors correlated with metabolic challenges, such as exercise^[18–19]^. Therefore, the role of PGC-1α in linking environmental requirements to metabolic demands and synchronizing these with the circadian rhythm is functionally important. Indeed, both SIRT1 and PGC-1α are involved in the control of the circadian rhythm period of the SCN clock, which has been confirmed in genetic studies. For example, it was shown that genetic ablation of *Sirt1* or pharmacological inhibition of SIRT1 in mice resulted in the disruption of circadian rhythms and the acetylation of histone H3 and BMAL1, which in turn elongated the circadian period^[20]^; mice that over-expressed SIRT1 in the brain were characterized by a shortened circadian period^[21]^; it was also concluded that the SIRT1/PGC-1α amplifying loop played a crucial role in the SCN pacemaker, being implicated in the remodeling of circadian chromatin and controlling rhythmicity in histone deacetylation^[20–21]^. These results also indicate that PGC-1α may be involved in the molecular clock entrainment by providing information about evolutionary advantages (such as physical exercise, caloric restriction, and cognitive demand).

## Entrainment in the circadian system

The biological clock generates circadian rhythm even in the absence of environmental synchronizers (in a free-running condition). However, there are environmental cues that can help the clock anticipate rhythmic geophysical and social processes, such as light/dark, temperature and humidity, food, and daily social activities. The mammalian master clock receives the most important entraining signal (the zeitgeber), determining physiology, biochemistry and behavior, from the changing light environment at dawn and dusk. Under the influence of this zeitgeber, the period of the SCN clock is synchronized to 24 h, and the phase of the clock corresponds to the current geophysical time. This is done primarily by photic entraining signals from the retina, which are transmitted from a subset of intrinsically photosensitive ganglion cells through the retinohypothalamic tract (RHT)^[22]^. These ganglion cells express melanopsin, a photo pigment with a peak sensitivity at around 480 nm (blue range of the light spectrum)^[23]^. RHT was found to end in a population of vasoactive intestinal peptide-producing neurons in the ventrolateral part of the SCN^[23]^. Other studies showed that this part of the SCN (the "core") contained an intrinsic pacemaking oscillator responsible for the synchronization of the circadian activity in the rest of the nucleus^[13,24]^. Notably, the SCN neuronal network was found to remain synchronized even in free-running conditions, when the external entraining signals were absent^[13]^. This provides a coherent circadian output signal of the SCN though in most species, a period of the intrinsic rhythm is slightly different from 24 h. That is why, a continuous synchronization of the intrinsic clock is required. Studies have concluded that the SCN is the most effectively entrained by light *via* RHT that can directly modify the timing, period, phase and amplitude of the SCN clock oscillations, because the light-derived signals influence daily expression patterns of CCGs and induce changes in numerous physiological, biochemical and behavioral rhythms^[25–26]^.

Apart from light, other cues like exercise, feeding, temperature, anxiety, and social events have been shown to serve as non-photic zeitgebers^[11,15,27–29]^. Analysis of the non-photic phase response curves showed that the curves for activity induced by cage changing, social interaction, and novel wheel were analogous in shape and displayed a high similarity to the dark pulse phase response curves under constant light in hamsters^[30]^. It was concluded that the activity-induced non-photic effects were opposite to the effects of light. Such a conclusion was supported by *in vivo* electrophysiology in nocturnal rodents displaying a suppression of the SCN neurons by behavioral activity^[31]^. The reduced spike activity in the SCN neurons may be produced by inhibitory neuropeptide Y projections from the intergeniculate leaflet or by inhibitory serotonergic projections from the raphe nuclei^[32–34]^. Circadian clocks in the periphery were found to be synchronized both by the SCN and by tissue-specific zeitgebers^[35]^. Being respectively independent, each peripheral clock adapts to its own external and internal stimuli, such as metabolic consequences of muscle activity or feeding cues, but is also sensitive to the light-dark cues entraining the master SCN clock. Thus, peripheral clocks respond to exercise or feeding schedules, enabling zeitgebers to optimize a fine temporal adjustment of specific metabolic reactions in the tissue. As compared with the master clock, peripheral clocks have been found to display a specific pattern of circadian phase shifts as a result of both, *i.e.*, indirect synchronization by light *via* the SCN and direct entrainment to peripheral signals^[15]^. Besides, studies confirmed that the synchronization of peripheral clocks by the SCN is mandatory, because the intrinsic rhythms of most peripheral oscillators gradually damp out in the absence of the synchronizing output from the SCN^[13,36]^.

In the muscle clock, contractile activities driven by motor neurons and feeding/fasting have served as dominant zeitgebers^[14,37–38]^. By regulation of CCGs responsible for anabolic and catabolic processes, the muscular clock provides a basis for the temporal separation of biochemical processes by setting the shifts in carbohydrate and lipid metabolism depending on the time of the day. In particular, it has been reported that the early active period of exercise is characterized by a peak in the expression of genes regulating glycolytic flux in the Krebs cycle, which induces a switch from lipid to carbohydrate catabolism; subsequently, at the end of the active phase of the exercise, there is an increase in the expression of genes responsible for lipogenesis and carbohydrate deposition, when postabsorptive storage of excess energy predominate; later, during the inactive period for the skeletal muscle, the genes responsible for fatty acid catabolism are activated, so lipids become a major source of energy^[39]^. Genetic studies have also confirmed the importance of the circadian rhythm of the muscle clock for homeostasis; for example, the muscle-specific *Bmal1* knockout mouse model was found to cause an impaired glucose uptake and metabolism^[37]^, indicating a crucial anticipatory role for the circadian clock in skeletal muscle at the sleep/wake transition, when glucose turns into the main source of energy for the skeletal muscle. In aggregate, studies have shown that the muscle molecular clock determines the circadian rhythm in expressing more than two thousand genes, including *PGC-1α* and *FNDC5*^[14,40–41]^.

## Irisin as an integrator of photic and non-photic entrainment

**Figure 2:**
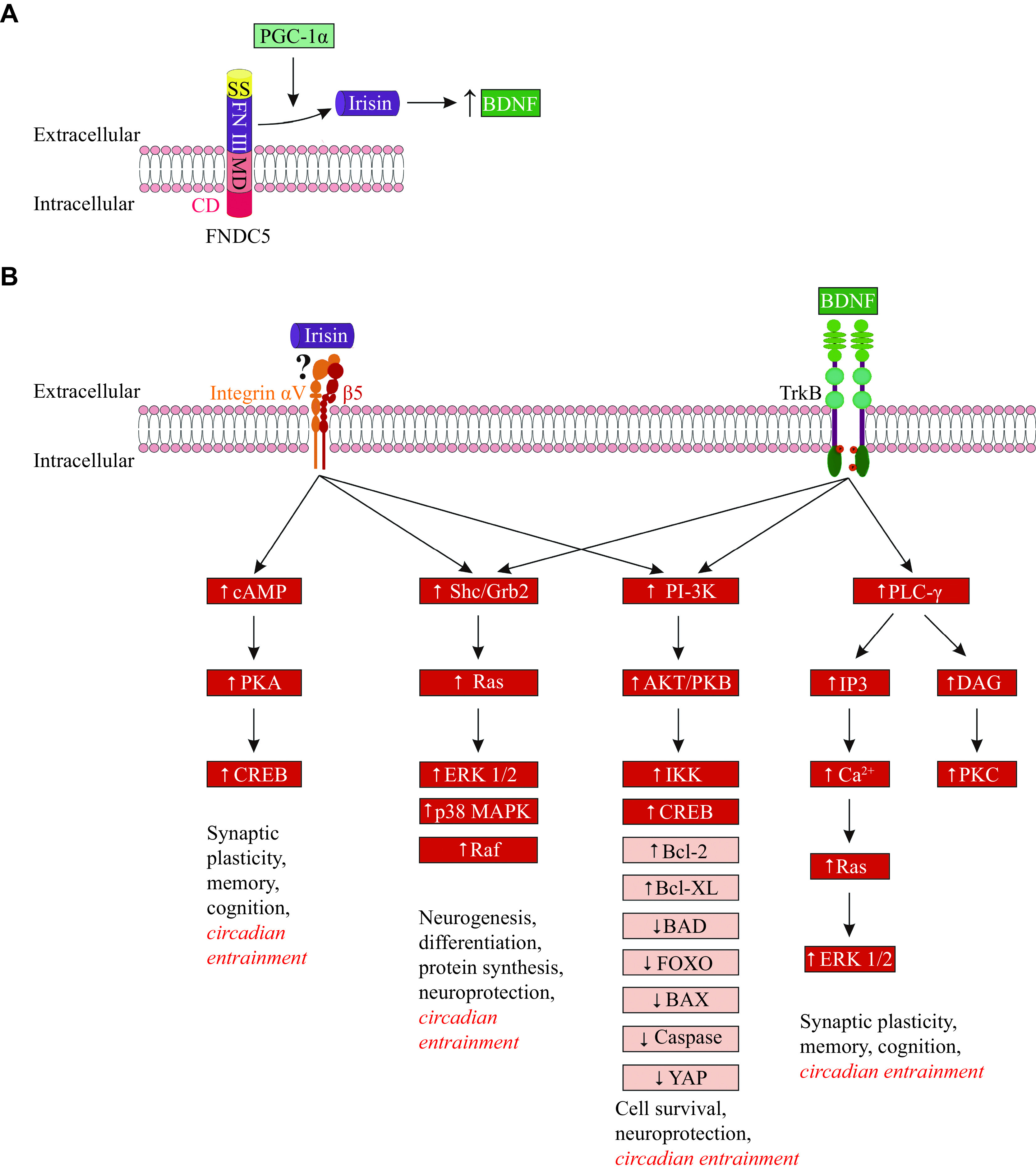
Cleavage of irisin from FNDC5, stimulation of BDNF expression by irisin, and irisin/BDNF signaling.

Irisin is a contraction-regulated myokine, cleaved from its parent FNDC5 (***Fig. 2A***) during motor activity, which induces the browning of white adipocytes. Irisin was found to be a product of cleavage and modification of the FNDC5 extracellular portion, although the suggested cleavage mechanism of irisin needs confirmation^[42]^. Irisin is a 12 kDa polypeptide with 100% conservation in the majority of mammals, assuming an evolutionary retained functionality^[43]^. As an irisin precursor, FNDC5 is known as a glycosylated membrane protein, controlled by PGC-1α. Being the principal regulating molecule for mitochondrial biogenesis, PGC-1α activates a set of downstream factors of the transcription. These factors, in particular, have been found to upregulate oxidative phosphorylation, replication/transcription of mitochondrial DNA, and import of mitochondrial proteins^[12,44]^, inducing the expression of anti-apoptotic proteins (*via* nuclear factor kappa B [NF-κB] and CREB), antioxidant and DNA repair enzymes, protein chaperones and Ca^2+^-regulating proteins^[45]^. The expression of PGC-1α and FNDC5 was shown to be activated by endurance exercise and cold^[46–48]^. Although the production of FNDC5 is mostly substantial in skeletal muscle, it was also demonstrated in various tissues and organs, *e.g.*, the adipose tissue, pericardium, heart, kidney, rectum, liver, lung, and brain^[43–44]^. Studies demonstrated that the proteolytic cleavage of irisin from its precursor FNDC5 was triggered by muscle contractions, and then irisin was released into the circulation^[2,4–5]^.

Irisin has both peripheral and central effects. The most eminent peripheral effect of irisin was reported to upregulate genes inducing thermogenesis, which was presumably mediated by p38 and ERK mitogen-activated protein kinases^[49]^. Furthermore, this effect was manifested in a metabolic switch to the browning of white fat, together with an elevated oxygen consumption and stimulation of thermogenesis in adipocytes^[2,46]^. In addition to its peripheral activity, irisin is able to pass through the blood-brain barrier (BBB) to display central effects^[42,50]^. In humans and rodents, FNDC5/irisin has been reported to be expressed in several regions of the brain, such as the hippocampus, midbrain, cerebellum, hypothalamus, cortex, and medulla oblongata^[4,51]^. In the hypothalamus, an intense expression of irisin was detected in a subset of the paraventricular nucleus that produced neuropeptide Y^[52]^. Irisin is also identified in the cerebrospinal fluid (CSF). It was reported that positive influence of endurance exercise might be mediated by FNDC5/irisin, as an upregulation of FNDC5 expression in the murine hippocampus was induced by exercise^[4,50,53]^.

In many brain areas (*e.g.*, the hippocampus, hypothalamus, frontal lobe, midbrain, and brain stem), an increase in PGC-1α levels induced by exercise was detected^[54]^. It was also shown that in the cultured hippocampal neurons, PGC-1α influenced the molecular machinery underlying the synaptogenic program and enhanced dendritic spine density, suggesting a pivotal role for PGC-1α in the development and adaptive modification of neural circuits^[55]^. This process is fundamental to neuronal plasticity and adaptation to environmental challenges. In *Pgc-1α*^−/−^ mice, FNDC5 expression was found to be reduced in both skeletal muscles and the brain, suggesting the possibility of serving PGC-1α as an upstream regulator of FNDC5 production^[4]^.

Irisin was found to induce the expression of BDNF in several regions of the brain such as the hippocampus, which was observed following the elevated peripheral irisin concentrations, as well as in primary hippocampal culture^[4–5]^. By contrast, a decreased BDNF expression in response to mRNA expression-mediated knockdown of *FBDC5* was evidenced^[4]^. It was reported that a single injection of irisin into the third brain ventricle caused an enhanced motor activity along with a reduction of resting time in rats^[48]^. Moreover, it was suggested that irisin released during the exercise could mediate muscle-brain crosstalk by linking the activity and circadian rhythms, thus providing the capacity to react adaptively to the environment^[56]^, which is consistent with the concept of entrainment of the SCN clock by exercise, and with the proposed involvement of irisin in the mechanism of entrainment^[11,15,56]^.

To produce coherent circadian oscillations, the master clock integrates information from environmental zeitgebers (photic information from the retina is the most important) as well as signals from the periphery^[57]^. It has been shown that under constant darkness, some peripheral synchronizing signals (*e.g.*, exercise) can phase shift the SCN clock^[11,30,58]^, suggesting that peripheral signalization per se can be potentially sufficient for entraining the master clock at least in the free-running conditions. Even under light/dark conditions, non-photic zeitgebers, such as scheduled exercise, restricted feeding schedule and hypocaloric feeding, have been found to entrain the SCN clock to a certain degree^[11,59]^. There are important pieces of evidence for the effects of muscular clock genes on behavioral circadian rhythms and sleep. For example, by utilizing the mice with an intact SCN neurochemical signaling as well as with a disrupted SCN neuropeptide signaling, investigators studied the effects of daily scheduled voluntary exercise on behavioral rhythms and SCN neuronal as well as molecular activities, and showed that in mice with the impaired neuropeptide signaling, scheduled exercise promoted the synchrony of the SCN cells and reinforced the 24-h rhythm in behavior; notably, the scheduled exercise reduced the activity of the SCN neurons and downregulated the cell-coupling opposing actions of GABAergic neurotransmission in both intact and neuropeptide-signaling deficient mice^[60]^. Moreover, one study also showed that several aspects of sleep were affected in the whole-body *Bmal1* knockout mice, whereas the rescue of *Bmal1* expression specifically in skeletal muscle (but not the brain) was sufficient to restore a normal amount of sleep in these mice^[61]^, concluding that *Bmal1* expression in skeletal muscle is both necessary and sufficient to regulate total sleep amount. Furthermore, it was found that the activity level correlated with the period length of rhythm in the master clock^[62–63]^. For example, in rodents, one study found that intensive voluntary locomotor activity (*e.g.*, wheel running) modulated phase responses, and bouts of wheel running might induce advanced or delayed phase shifts depending on circadian time^[64–65]^. Another study on golden hamsters showed that physical activity on the subjective day caused a phase advance, so the animals woke up and went to sleep earlier^[66]^. In humans, similar effects of physical activity bouts were described^[67]^, which showed that motor activity could elicit phase shifts of the melatonin rhythm varying with a circadian phase of exercise exposure; the exercise also caused phase advance when given in the early evening, while nocturnal activity might delay the phase of melatonin rhythm^[27,68–69]^. Furthermore, the timed physical exercise was found to accelerate re-entrainment of the human daily cycle to an 8-h phase-advanced schedule^[70]^. Thus, for a better entraining of the SCN clock, the use of combined light and exercise is preferable. In adolescents suffering from a delayed circadian rhythm, to induce phase advance of the SCN clock, current "gold standard treatments" (*i.e.*, morning light therapy and evening melatonin administration) have been recommended to combine with early morning exercise^[27,71]^. It was noted that exercise in the morning was able to improve well-being, enhance mood, reduce daytime hypersomnia, and improve cognitive functions^[72–73]^. Besides, time-restricted feeding is also used to entrain the SCN clock. One study found that a time-restricted feeding at ZT0-4 (zeitgeber time) produced a significant long-term delay of the activity offset in mice^[74]^, further indicating an important role for the insulin-like growth factor 2 (IGF2)/potassium chloride cotransporter 2 (KCC2) signaling in these behavioral changes. It is also suggested that the SIRT1/PGC-1α amplifying loop is involved in the entraining effect of restricted feeding^[56]^, which involves the activation of PGC-1α by stimulation of free fatty acid and β-hydroxybutyrate synthesis, or SIRT1-associated modulation of the NADH/NAD^+^ ratio^[17,75]^. It was hypothesized that PGC-1α, either by local production or by the induction of irisin expression, might increase BDNF levels to the amount sufficient for the facilitation of glutamate transmission and entrainment of the SCN clock^[56]^.

One study with the mice model of jet lag induced by shifted light cycles revealed that resynchronization to the new time zone could be accelerated by exercise^[76]^. Other studies in rodents also indicated that motor activity could stabilize the circadian rhythms, in particular, in golden hamsters; it was shown that the wheel running could synchronize the circadian rhythm of body temperature^[77]^, and this effect was supported by the data obtained in aging mice, in which it was found that previously highly variable onset of activity became stronger coupled to the end of the light period, in the presence of a running wheel^[78]^. Thus, it has been concluded that the enhanced amount of motor activity per day can improve the stability and synchronization of the circadian rhythmicity in aging mice^[79]^. Moreover, studies in Djungarian hamsters, when developing a delayed daily activity onset or an arrhythmic phenotype under standard laboratory conditions, found that the running wheel activity stabilized photic entrainment^[63,80]^; in these experimental conditions, a wild-type activity, characterized by its onset tightly corresponding to the beginning of the light phase, was eventually developed when animals were given access to running wheels; the authors could not explain this phenomenon by changes in endogenous period length or photic phase response, but concluded that mechanisms downstream from the SCN were involved^[80]^.

Characterizing the entraining mechanism of irisin, the possible effect of hyperthermia, induced by exercise, needs special attention. Studies in humans have shown that heat stimulation increases the levels of circulating irisin^[81]^, and that the entrainment of circadian rhythm in the cultured murine SCN cells has 1.5 ℃ cycles of temperature^[82]^. In another *in vitro* study, simulated body temperature cycles gradually synchronized circadian gene expression in cultured fibroblasts of both mice and humans^[83]^. It was revealed that heat-shock factor 1 (HSF1) was required for the efficient synchronization of fibroblast oscillators^[83]^. Thus, theoretically, an increase in body temperature (accompanied by irisin production) can mediate the effects of physical exercise on the circadian phase, but it is not clear to what extent these results can be applied to in an *in vivo* situation. Future studies aiming at a comparative characterization of irisin and temperature-entraining effects *in vivo* are needed. It has been shown recently that extracellular heat shock protein 90α (Hsp90α) is secreted by muscle with exercise in mice, activating the putative irisin receptor, integrin αVβ5, and highly increasing its affinity for irisin (with a *Kd* of approximately 30 nmol/L)^[84]^, which demonstrates a possible ligand-receptor mechanism by which irisin probably mediates its physiological effects.

## The concept of a muscle-brain axis

Experimental studies have confirmed what scientists have long expected: exercise has a number of positive effects on lifespan, including inhibition of osteoporosis, myocardial ischemia, metabolic syndrome, and type 2 diabetes^[85–88]^. Besides, exercise has been shown to increase brain conditions to improve cognitive functions, dementia, depression, and sleep^[89–92]^. It was suggested that the positive influence of motor activity on cognitive functions might be caused by hippocampal upregulation of BDNF production^[93]^. In 2011, Norheim *et al*^[94]^ found that athletic training induced an enhanced transcription of secretory proteins, and more than 10 myokines, including cathepsins, in muscles. For example, complex studies in humans, mice, and monkeys revealed an increased level of cathepsin B in blood plasma and its ability to pass the BBB after running; furthermore, an increased production of BDNF, induced by cathepsin B, was recorded in the hippocampus, suggesting a promoting action of BDNF on neurogenesis, learning, and memory^[95]^. In the long-term (35 [± 15] years) exercise-trained humans, an improved memory along with the reduced levels of resting serum BDNF, cathepsin B, malondialdehyde, and index of lipid peroxidation were found^[96]^. At present, FNDC5/irisin has been considered the potent exercise-induced modulator of brain functions. PGC-1α and FNDC5/irisin are widely expressed in the brain. Studies have indicated an improvement of several brain functions by exercise *via* BDNF upregulation^[4,50–52,54,97]^. In rodents, the *Fndc5* mRNA has been found in the midbrain, pons, cerebellum, olfactory bulb, hippocampus, and cortical neurons, where neuronal *Fndc5* gene expression is mediated by PGC-1α^[4,50,98]^. Endurance exercise was reported to induce an enhanced FNDC5 production in the murine hippocampus^[4,50]^. Another study found that eight weeks of exercise training in mice resulted in a significant increase in *Pgc-1α* mRNA expression (up to 3-fold) in the brain stem, cortex, frontal lobe, hippocampus, hypothalamus, and midbrain^[54]^, consistent with the regulation of BDNF production by FNDC5 and PGC-1α in neurons. Thus, the positive action of endurance exercise on cognitive functions may be mediated by FNDC5/irisin^[53]^, as directly evidenced by the delivery of FNDC5 to the liver *via* an adenoviral vector, resulting in the elevated level of blood irisin and enhanced expression of the *Bdnf* gene in the murine hippocampus^[4]^.

There are indications of the beneficial influence of FNDC5/irisin on brain functions in Alzheimer's disease (AD). For example, Lourenco *et al*^[98]^ found that hippocampal long-term potentiation (LTP) disappeared after injections of hairpin-*Fndc5* RNA into mouse brains; similarly, the defects of memory and behavior and the failure of LTP occurred in an experimental AD model caused by the injection of amyloid-β oligomers (AβOs), but when recombinant irisin was administered along with AβOs, these behavioral deficits and LTP were reversed^[98]^. Moreover, in this study, the behavioral defects induced by AβOs were also reversed by exercise and viral transfection with *Fndc5*; subsequently, FNDC5/irisin overexpression rescued the impaired memory of the AD mice. By contrast, the positive neuroprotective effects of motor activity were attenuated by the blockade of FNDC5/irisin either in the periphery or in the brain of the mice^[98]^. Since peripheral irisin is able to modulate brain functions, it is reasonable to suggest that the BBB is permeable for irisin, but the detailed mechanism of a possible irisin transport is still obscure^[42]^. Some studies suggest a central origin of irisin. In one study of elderly men, quantitative mass spectrometry with the labeled peptides was employed to measure irisin in CSF^[99]^, and irisin concentration in CSF was found to be 0.26–1.86 ng/mL, but it was not identified in the plasma, suggesting that the origin of CSF irisin might be the brain FNDC5^[42]^. Still, a peripheral origin of irisin presenting in CSF is more probable. For example, one study compared the circulating and CSF irisin levels in pregnant women, and found that irisin levels was approximately 20- to 25-fold lower in the CSF than in the circulating serum; however, a positive linear correlation between serum and CSF irisin levels was also observed, suggesting a predominantly peripheral origin of irisin and indicating that the BBB limited the access of irisin to the brain^[52]^. The BBB can block the transfer of proteins and other large molecules from plasma to the CSF, but the block is not complete, because occasionally small quantity of proteins is able to enter the brain by diffusion. As irisin plays a significant role in brain physiology, it seems more likely for irisin to have a selective BBB transporter or a high-affinity brain receptor. Besides, an alternative possibility to improve learning and memory was proposed by EI Hayek *et al*^[100]^, in which exercise promoted an elevation of lactate levels in circulation, then lactate entered hippocampal neurons through the monocarboxylate transporter 2 (MCT2) and induced a subsequent activation of Sirt1, PGC-1α, FNDC5, and BDNF. This proposed mechanism outflanks the need to penetrate the BBB for irisin, but it remains to be experimentally confirmed.

## The irisin receptor: an unexplored element of the muscle-brain axis

In an early study, the existence of a cell surface receptor mediating effects of irisin was hypothesized^[2]^. Unfortunately, till the present, the receptor for irisin has not been completely identified. In 2018, it was first reasoned that osteocytes must express such a receptor, as osteocytes in culture responded to the recombinant irisin by upregulating a local modulator of bone remodeling, sclerostin, and by an increased survival of osteocytes^[101]^. One study performed transfection of HEK 293T cells with various integrins, and found that the best response to recombinant irisin was in cells with αVβ5 integrin, which was concluded to be a likely irisin receptor^[101]^. Specifically, in osteocytes and fat cells, the signaling pathway activated by irisin was blocked by chemical inhibition of the αV integrins, indicating that an unidentified irisin receptor possibly exerts its action *via* αVβ5 integrin^[101]^. Subsequent studies supported αVβ5 as a putative irisin receptor^[102–104]^. Particularly, it was shown that intestinal epithelial barrier dysfunction in ischemia could be reversed by irisin *via* its binding to integrin αVβ5^[102]^. Although it was observed that irisin stimulated murine bone resorption acting on osteoclast progenitors, this effect of irisin was prevented by a neutralizing antibody to integrin αVβ5^[104]^. Besides, in a study on fat tissue, a unique type of adipocyte progenitor cells (APCs) was described, which possessed an ability to initiate formation of beige adipocytes; these proliferative beige APCs expressed surface proteins, such as tetraspanin CD81; for the proliferation of beige APCs, a complex of CD81 with αVβ1 and αVβ5 integrin was required; further, this CD81 controlled integrin-FAK (focal adhesion kinase) signaling in response to irisin^[103]^. These demonstrate the existence of cell surface receptors for irisin as it has been foreseen, and αVβ5 integrin is supposed to be the cellular receptor for irisin in osteocytes, adipocytes, and enterocytes. However, in other peripheral cell types, the irisin receptor still needs to be identified.

The existence of functional irisin receptors in the CNS is also postulated. For example, Lourenco *et al*^[98]^ demonstrated that irisin was capable of opposing synapse loss and amnesia in mouse models of AD; this *in vitro* study suggested that FNDC5/irisin could bind to the unexplored receptors located on the membrane of hippocampal neurons and astrocytes to mediate their beneficial effects. Regarding this evidence, there was a suggestion that the binding of FNDC5/irisin to its putative CNS receptor initiated internalization of the receptor into the cytoplasm by endocytosis; however, this is still unclear because the receptor has not been characterized^[51]^. The future discovery of the irisin receptor in the brain will have a potential therapeutic relevance, and it will provide a possibility to identify specific mechanisms underlying the protective effects of irisin.

There have been several proposed intracellular pathways involved in the transduction of irisin effects. The major pathways suggested to mediate central effects of irisin, including circadian entrainment, are the MAPK/ERK pathway, the PI3K/AKT pathway, and the cAMP/PKA/CREB pathway (***Fig. 2B***). Unfortunately, to date, it is only possible to speculate that the similar FNDC5/irisin receptor, which has been characterized in the bone and adipose tissues, is expressed in the brain. With that, many possible pharmacological interventions can be applied. It needs to be noted that although αVβ5 integrin has been shown as a putative irisin receptor, there is still a possibility for other receptors^[105]^. For a better characterization of irisin mechanisms, the identification of its receptor is required.

## BDNF controls the access for photic input in the SCN

BDNF, belonging to classical family of neurotrophins, is synthesized in a form of prepro-neurotrophin, which is converted into pro-BDNF and then transformed into mature BDNF by a plasminogen activator^[106]^. The BBB is permeable for BDNF, which produces peripheral and central effects, often through the activation of genes coding regulatory proteins of cell energetic metabolism, mitochondrial biogenesis, synaptic plasticity, and cell survival^[106]^. Thus, BDNF is actively involved in the development, maintenance and plasticity of the nervous system^[107]^. BDNF displays a significant synaptic activity. By altering N-methyl-D-aspartic acid (NMDA) receptor activation kinetics and increasing the vesicular docking and fusion, BDNF can promote synaptic transmission, triggering LTP and adaptive transformation of neuronal circuity^[55,108]^. BDNF has also been shown to promote dendritic spinogenesis in hippocampal CA1 neurons, enhancing the structural plasticity of dendrites in the dentate gyrus^[55]^.

One study showed that BDNF was stored in vesicles that were actively transported along the axon to presynaptic terminals and dendrites, from where it was released after the activation of the glutamate receptor^[109]^. Two types of receptors are specific for the released BDNF: the high-affinity tropomyosin-related kinase receptor B (TrkB) receptor and the low-affinity p75 neurotrophin receptor (p75NTR). The binding of BDNF to TrkB was shown to engage phospholipase C gamma (PLC-γ), phosphatidylinositol-3 kinase (PI3K), and MAPK/ERK intracellular signaling (***Fig. 2B***), enhancing transcription factors that induced the expression of neurochemical regulators involved in BDNF central effects^[107]^. Specifically, studies showed that BDNF prevented apoptosis in neuronal cells by inhibiting the production of pro-apoptotic proteins, such as BAX and BAD, and by activating the production of anti-apoptotic proteins (BCL-2 family) and caspase inhibitors; besides, BDNF up-regulated antioxidant enzymes and protected neurons from oxidative DNA damage through mechanisms involving enhanced DNA repair^[107,110]^. BDNF was also reported to be responsible for neurite outgrowth and synaptogenesis, promoting both excitatory and inhibitory neurotransmission *via* the activation of p21 Ras, and playing a prominent role in the establishment of neural circuit architecture, including circuits regulating energy homeostasis^[111–112]^. Additionally, BDNF was found to be involved in the regulation of circadian behavioral and neuroendocrine patterns^[106]^. Besides, BDNF was shown to upregulate PGC-1α, a stimulator of mitochondrial biogenesis; conversely, PGC-1α increased the expression of BDNF, thus providing a bidirectional positive feedback mechanism^[4]^.

BDNF is widely distributed in the brain, localized particularly in the hippocampus, prefrontal cortex, amygdala, and ventral tegmental region^[113]^. These brain areas are known to be involved in reward-related reinforcement learning that leads to behavioral changes^[114]^. High levels of BDNF and its main receptor TrkB have been found across hypothalamic nuclei, including the SCN^[115–116]^. BDNF has been suggested to mediate beneficial effects of exercise and intermittent fasting on cognitive functions^[53]^. Indeed, an increased expression of BDNF in many brain areas accompanied by an improved cognition was demonstrated, because of the effects of voluntary aerobic exercise and intermittent fasting^[117–119]^. This is also in line with an upregulation of BDNF expression by irisin during exercise in another study^[4]^. In addition to irisin, neuropeptides and excitatory synaptic neurotransmitters were also found to mediate the expression and release of BDNF^[106,120]^. In this regard, the sequence of events induced by glutamate, the major excitatory neurotransmitter, was indicative; the activation of NMDA receptors by glutamate increased intracellular Ca^2+^ concentrations, enhanced activities of protein kinase C (PKC), Ca^2+^/calmodulin-dependent protein kinase (CaMK), and ERK, which in turn stimulated CREB and transcription factor NF-κB, inducing the transcription of the *Bdnf* and the following loading of BDNF into the synaptic vesicles; further, BDNF was released from vesicles and bound to its cognate tropomyosin kinase receptor, TrkB^[106]^.

BDNF signaling is implicated in the regulation of circadian rhythms. The circadian pattern of BDNF expression was shown in the SCN, with peak levels during subjective night, suggesting that BDNF signaling should be insufficient to release excitatory transmitter and to transmit the photic signal through the RHT-SCN synapse during subjective daytime; in contrast, the increased levels of BDNF at subjective night might be sufficient to mediate the light-induced activation of the SCN through the RHT^[121]^. There is also some evidence in rats showing that exogenous BDNF injected directly into the SCN during subjective afternoon (a period of insensitivity of the master clock to light) induced the prominent phase advance of the free-running rhythm in response to light exposure; in contrast, during subjective night, BDNF did not affect phase-shifting effects of light^[122]^. These results suggest that exogenous BDNF is able to shift the circadian oscillator only during the subjective day when levels of endogenous BDNF are low. Furthermore, in the *Bdnf^+/−^* mice, a decreased amplitude of phase shifts, induced by light exposure during subjective night, was demonstrated, and tyrosine kinase inhibitors, directly administered into the SCN, suppressed phase-shifting effects of light during subjective night^[122]^. Importantly, the BDNF/TrkB signaling alone was found to be sufficient to produce a phase shift in the SCN spike activity rhythm *in vitro*^[123–124]^. Thus, it is hypothesized that BDNF plays a key role in the photic entrainment of the SCN clock by mediating phase shifts.

As an intracellular membrane-anchored G protein, Ras has been identified as an upstream element in the ERK signaling pathway of the SCN clock and an important effector of BDNF signaling^[125]^. The modulation of Ras activity has been found to alter the photoentrainment of the clock and tune the circadian period length^[126]^. Electron microscopy investigation showed the impaired day/night variations in the dendritic enwrapped the vasoactive intestinal polypeptide-expressing neurons by astrocytic processes (glial coverage), because of the inhibition of the TrkB signaling, suggesting the involvement of BDNF-mediated structural changes of the SCN in the photic entrainment^[127]^. Furthermore, the terminals of RHT fibers in the SCN express synaptic TrkB receptors were in close proximity to BDNF-expressing SCN neurons, suggesting the involvement of BDNF in the modulation of synaptic transmission^[123–124]^. BDNF was also found to stimulate the synaptic release of neurotransmitters, glutamate and PACAP^[123–125]^. Besides, BDNF was reported to potentiate the postsynaptic response to glutamate, through the phosphorylation of NMDA receptor and the following increased probability of channel opening, or through the accelerated cycling of NMDA receptor to increase the amount of the membrane NMDA receptors^[124,128–129]^. It has been reported that the increased presynaptic glutamate release, induced by BDNF, is modulated by the activation of TrkB receptors^[130–131]^.

**Figure 3:**
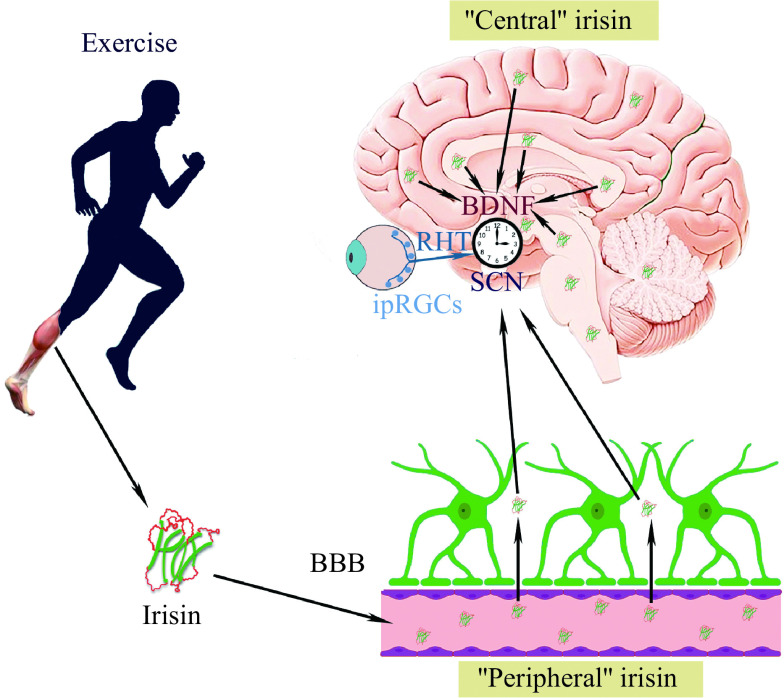
A schematic outline of irisin's action on the SCN master clock.

Taken together, the available studies suggest that irisin, either penetrating the BBB from systemic circulation, or by its direct expression in the brain, increases BDNF levels in the retinohypothalamic synapse and that irisin may modulate the effect of BDNF on photic entrainment (***Fig. 3***). However, when discussing a role for irisin/BDNF in circadian function, the following considerations should be taken into account. First, a large amount of data is obtained from *in vitro* experiments, thereby it is necessary to keep in mind that the stressed neurons (because of slice preparation, microinjections and other manipulations) may non-specifically produce plasminogen activator^[132]^, in turn inducing expression of BDNF. Beyond this, irisin is not the only regulator of the BDNF expression. For example, in the pain neural circuit, a key role in the regulation of BDNF expressions was found to be the WNT/β-catenin signaling^[133]^. If this mechanism is correlated with the circadian function, it cannot be excluded that not all BDNF effects are associated with irisin, so irisin and BDNF circadian activities may be dissociated to a certain extent. Notably, the link between irisin and BDNF in the SCN is still considered hypothetical^[56]^.

We conclude that BDNF may play an essential role in the regulation of SCN pacemaker sensitivity to light, as BDNF is involved in multiple aspects of the circadian pattern of physiological, behavioral, and neuroendocrine processes correlated with energy homeostasis. The BDNF/TrkB signaling is involved in synaptic transmission between the RHT and the core of the SCN, modulating the presynaptic release of glutamate and the postsynaptic response of NMDA receptors to glutamate. It is thought that BDNF is required for glutamate to activate cascades, thus transducing light signals to the SCN and altering the phase of the clock. Therefore, BDNF plays a critical role in photoentrainment by triggering the entry of light-related input into the SCN^[56]^.

## Conclusions

In summary, as a contraction-regulated myokine, irisin plays a prominent role in the circadian system. Within the muscle-brain axis, non-photic entraining signals fine-tune the SCN circadian clock by irisin released into the circulation. Irisin is able to enter the blood by crossing the BBB, and the expression of irisin in several brain regions is also shown. Irisin facilitates the SCN entrainment to light *via* BDNF increasing its release from synaptic terminals of the RHT. To facilitate photic entrainment, sufficiently high levels of BDNF are required, and BDNF renders as the gatekeeper for the master clock entrainment to light. Thus, irisin is a key player in facilitating photic entrainment *via* BDNF, and in the integration of photic and non-photic entrainment of the SCN in response to exercise. The cellular receptor for irisin remains to be identified, which will greatly facilitate a better understanding of irisin's function in exercise and human health.

## References

[b1] 1Legård GE, Pedersen BK. Muscle as an endocrine organ[M]//Zoladz JA. Muscle and Exercise Physiology. London: Academic Press, 2019: 285–307.

[2] (2012). A PGC1-α-dependent myokine that drives brown-fat-like development of white fat and thermogenesis. Nature.

[3] (2012). Beige adipocytes are a distinct type of thermogenic fat cell in mouse and human. Cell.

[4] (2013). Exercise induces hippocampal BDNF through a PGC-1α/FNDC5 pathway. Cell Metab.

[5] (2015). FNDC5/Irisin - their role in the nervous system and as a mediator for beneficial effects of exercise on the brain. Brain Plast.

[6] (2008). Two decades of circadian time. J Neuroendocrinol.

[7] (2014). A circadian gene expression atlas in mammals: implications for biology and medicine. Proc Natl Acad Sci U S A.

[8] (2018). Diurnal transcriptome atlas of a primate across major neural and peripheral tissues. Science.

[9] (2005). Come together, right...now: synchronization of rhythms in a mammalian circadian clock. Neuron.

[10] (2015). Circadian rhythm phase shifts and endogenous free-running circadian period differ between African-Americans and European-Americans. Sci Rep.

[11] (2017). The mammalian circadian clock and its entrainment by stress and exercise. J Physiol Sci.

[12] (2007). Transcriptional coactivator PGC-1α integrates the mammalian clock and energy metabolism. Nature.

[13] (2010). Suprachiasmatic nucleus: cell autonomy and network properties. Annu Rev Physiol.

[14] (2015). Circadian rhythms, the molecular clock, and skeletal muscle. J Biol Rhythms.

[15] (2018). Entrainment of the mouse circadian clock: effects of stress, exercise, and nutrition. Free Radic Biol Med.

[16] (2011). Age-associated disruption of molecular clock expression in skeletal muscle of the spontaneously hypertensive rat. PLoS One.

[17] (2013). Effects of resveratrol and SIRT1 on PGC-1α activity and mitochondrial biogenesis: a reevaluation. PLoS Biol.

[18] (1998). A cold-inducible coactivator of nuclear receptors linked to adaptive thermogenesis. Cell.

[19] (2007). Mitochondrial-nuclear communications. Annu Rev Biochem.

[20] (2008). The NAD^+^-dependent deacetylase SIRT1 modulates CLOCK-mediated chromatin remodeling and circadian control. Cell.

[21] (2013). SIRT1 mediates central circadian control in the SCN by a mechanism that decays with aging. Cell.

[22] (2022). Photic entrainment of the circadian system. Int J Mol Sci.

[23] (2021). Comparative neurology of circadian photoreception: the retinohypothalamic tract (RHT) in sighted and naturally blind mammals. Front Neurosci.

[24] (2001). Cellular communication and coupling within the suprachiasmatic nucleus. Chronobiol Int.

[25] (2011). Acute light exposure suppresses circadian rhythms in clock gene expression. J Biol Rhythms.

[26] (2004). Dark pulse resetting of the suprachiasmatic clock in Syrian hamsters: behavioral phase-shifts and clock gene expression. Neuroscience.

[27] (2017). Can exercise regulate the circadian system of adolescents? Novel implications for the treatment of delayed sleep-wake phase disorder. Sleep Med Rev.

[28] (2017). Ultradian feeding in mice not only affects the peripheral clock in the liver, but also the master clock in the brain. Chronobiol Int.

[b29] 29Wams EJ, Riede SJ, van der Laan I, et al. Mechanisms of non-photic entrainment[M]//Kumar V. Biological Timekeeping: Clocks, Rhythms and Behaviour. New Delhi: Springer, 2017: 395–404.

[30] (1989). Effects of induced wheel running on the circadian activity rhythms of Syrian hamsters: entrainment and phase response curve. J Biol Rhythms.

[31] (2012). Amplitude of the SCN clock enhanced by the behavioral activity rhythm. PLoS One.

[32] (2013). The NPY intergeniculate leaflet projections to the suprachiasmatic nucleus transmit metabolic conditions. Neuroscience.

[33] (2017). Effects of neuropeptide Y on neuron spike activity in the rat suprachiasmatic nucleus *in vitro*. Neurosci Behav Physiol.

[34] (2014). Exercise and sleep in aging: emphasis on serotonin. Pathol Biol.

[35] (2012). Advances in understanding the peripheral circadian clocks. FASEB J.

[36] (2000). Resetting central and peripheral circadian oscillators in transgenic rats. Science.

[37] (2016). The functional significance of the skeletal muscle clock: lessons from *Bmal1* knockout models. Skelet Muscle.

[38] (2013). Circadian rhythms, skeletal muscle molecular clocks, and exercise. Exerc Sport Sci Rev.

[39] (2015). The endogenous molecular clock orchestrates the temporal separation of substrate metabolism in skeletal muscle. Skelet Muscle.

[40] (2013). CircaDB: a database of mammalian circadian gene expression profiles. Nucleic Acids Res.

[41] (2017). The role of the molecular clock in skeletal muscle and what it is teaching us about muscle-bone crosstalk. Curr Osteoporos Rep.

[42] (2021). Progress and challenges in the biology of FNDC5 and irisin. Endocr Rev.

[43] (2013). FNDC5/irisin is not only a myokine but also an adipokine. PLoS One.

[44] (2018). The role of exercise-induced myokines in regulating metabolism. Arch Pharm Res.

[45] (2018). Intermittent metabolic switching, neuroplasticity and brain health. Nat Rev Neurosci.

[46] (2008). The role of exercise and PGC1α in inflammation and chronic disease. Nature.

[47] (2012). Expression of the irisin precursor FNDC5 in skeletal muscle correlates with aerobic exercise performance in patients with heart failure. Circ Heart Fail.

[48] (2015). Irisin: a myokine with locomotor activity. Neurosci Lett.

[49] (2014). Irisin stimulates browning of white adipocytes through mitogen-activated protein kinase p38 MAP kinase and ERK MAP kinase signaling. Diabetes.

[50] (2014). Neuroprotective effects of physical activity on the brain: a closer look at trophic factor signaling. Front Cell Neurosci.

[51] (2021). Multiple roles in neuroprotection for the exercise derived myokine Irisin. Front Aging Neurosci.

[52] (2014). The identification of irisin in human cerebrospinal fluid: influence of adiposity, metabolic markers, and gestational diabetes. Am J Physiol Endocrinol Metab.

[53] (2016). Does PGC1α/FNDC5/BDNF elicit the beneficial effects of exercise on neurodegenerative disorders?. Neuromolecular Med.

[54] (2011). Exercise training increases mitochondrial biogenesis in the brain. J Appl Physiol.

[55] (2012). Involvement of PGC-1α in the formation and maintenance of neuronal dendritic spines. Nat Commun.

[56] (2018). Blind spot for sedentarism: redefining the diseasome of physical inactivity in view of circadian system and the irisin/BDNF axis. Front Neurol.

[57] (2010). The mammalian circadian timing system: organization and coordination of central and peripheral clocks. Annu Rev Physiol.

[58] (1996). Entrainment and phase shifting of circadian rhythms in mice by forced treadmill running. Physiol Behav.

[59] (2012). Voluntary scheduled exercise alters diurnal rhythms of behaviour, physiology and gene expression in wild-type and vasoactive intestinal peptide-deficient mice. J Physiol.

[60] (2021). Timed daily exercise remodels circadian rhythms in mice. Commun Biol.

[61] (2017). *Bmal1* function in skeletal muscle regulates sleep. eLife.

[62] (1997). A nonlinear interrelationship between period length and the amount of activity—Age-dependent changes. Biol Rhythm Res.

[63] (2007). An inbred lineage of Djungarian hamsters with a strongly attenuated ability to synchronize. Chronobiol Int.

[64] (2007). Non-photic modulation of phase shifts to long light pulses. J Biol Rhythms.

[65] (2002). Djungarian hamsters: a species with a labile circadian pacemaker? Arrhythmicity under a light-dark cycle induced by short light pulses. J Biol Rhythms.

[66] (1996). Locomotor activity and non-photic influences on circadian clocks. Biol Rev.

[67] (2019). Human circadian phase-response curves for exercise. J Physiol.

[68] (2003). Exercise elicits phase shifts and acute alterations of melatonin that vary with circadian phase. Am J Physiol Regul Integr Comp Physiol.

[69] (1997). Roles of intensity and duration of nocturnal exercise in causing phase delays of human circadian rhythms. Am J Physiol.

[70] (2010). Physical exercise accelerates reentrainment of human sleep-wake cycle but not of plasma melatonin rhythm to 8-h phase-advanced sleep schedule. Am J Physiol Regul Integr Comp Physiol.

[71] (2022). Low-intensity scheduled morning exercise for adolescents with a late chronotype: a novel treatment to advance circadian phase?. Sleep Adv.

[72] (2012). Daily morning running for 3 weeks improved sleep and psychological functioning in healthy adolescents compared with controls. J Adolesc Health.

[73] (2020). Physical exercise during the morning school-break improves basic cognitive functions. Mind Brain Educ.

[74] (2022). Time-restricted feeding entrains long-term behavioral changes through the IGF2-KCC2 pathway. iScience.

[75] (2018). Circadian rhythms in mitochondrial respiration. J Mol Endocrinol.

[76] (2018). Two coupled circadian oscillators are involved in nonphotic acceleration of reentrainment to shifted light cycles in mice. J Biol Rhythms.

[77] (1994). Statistical and dynamical analysis of circadian rhythms. J Theor Biol.

[78] (2013). Voluntary exercise can strengthen the circadian system in aged mice. Age.

[79] (2000). Age-dependent changes of the circadian system. Chronobiol Int.

[80] (2018). Voluntary exercise stabilizes photic entrainment of djungarian hamsters (*Phodopus sungorus*) with a delayed activity onset. Chronobiol Int.

[81] (2021). Effect of heat stimulation on circulating irisin in humans. Front Physiol.

[82] (2003). Circadian entrainment to temperature, but not light, in the isolated suprachiasmatic nucleus. J Neurophysiol.

[83] (2012). Simulated body temperature rhythms reveal the phase-shifting behavior and plasticity of mammalian circadian oscillators. Genes Dev.

[84] (2023). Irisin acts through its integrin receptor in a two-step process involving extracellular Hsp90α. Mol Cell.

[85] (2005). Effects of physical activity on life expectancy with cardiovascular disease. Arch Intern Med.

[86] (2015). Exercise and regulation of adipokine and myokine production. Prog Mol Biol Transl Sci.

[87] (2017). Lifestyle recommendations for the prevention and management of metabolic syndrome: an international panel recommendation. Nutr Rev.

[88] (2018). An update on the role of cardiorespiratory fitness, structured exercise and lifestyle physical activity in preventing cardiovascular disease and health risk. Prog Cardiovasc Dis.

[89] (2010). Aerobic exercise and neurocognitive performance: a meta-analytic review of randomized controlled trials. Psychosom Med.

[90] (2019). Physical activity and exercise as a universal depression prevention in young people: a narrative review. Early Interv Psychiatry.

[91] (2019). Physical activity and muscle-brain crosstalk. Nat Rev Endocrinol.

[92] (2020). Effects of physical activity programs on sleep outcomes in older adults: a systematic review. Int J Behav Nutr Phys Act.

[93] (2007). Exercise builds brain health: key roles of growth factor cascades and inflammation. Trends Neurosci.

[94] (2011). Proteomic identification of secreted proteins from human skeletal muscle cells and expression in response to strength training. Am J Physiol Endocrinol Metab.

[95] (2016). Running-induced systemic cathepsin B secretion is associated with memory function. Cell Metab.

[96] (2019). Long-term exercise training improves memory in middle-aged men and modulates peripheral levels of BDNF and Cathepsin B. Sci Rep.

[97] (2021). Irisin: a new code uncover the relationship of skeletal muscle and cardiovascular health during exercise. Front Physiol.

[98] (2019). Exercise-linked FNDC5/irisin rescues synaptic plasticity and memory defects in Alzheimer's models. Nat Med.

[99] (2018). Detection and quantitation of irisin in human cerebrospinal fluid by tandem mass spectrometry. Peptides.

[100] (2019). Lactate mediates the effects of exercise on learning and memory through SIRT1-dependent activation of hippocampal brain-derived neurotrophic factor (BDNF). J Neurosci.

[101] (2018). Irisin mediates effects on bone and fat *via* αV integrin receptors. Cell.

[102] (2020). Irisin reverses intestinal epithelial barrier dysfunction during intestinal injury *via* binding to the integrin αVβ5 receptor. J Cell Mol Med.

[103] (2020). CD81 controls beige fat progenitor cell growth and energy balance *via* FAK signaling. Cell.

[104] (2020). Irisin directly stimulates osteoclastogenesis and bone resorption *in vitro* and *in vivo*. eLife.

[105] (2022). FNDC5/Irisin: physiology and pathophysiology. Molecules.

[106] (2014). BDNF mediates adaptive brain and body responses to energetic challenges. Trends Endocrinol Metab.

[107] (2006). Neurotrophin signalling in health and disease. Clin Sci (Lond).

[108] (2008). Serum brain-derived neurotrophic factor levels in patients with major depression: effects of antidepressants. J Psychiatr Res.

[109] (2012). Distinct subsets of Syt-IV/BDNF vesicles are sorted to axons versus dendrites and recruited to synapses by activity. J Neurosci.

[110] (2014). BDNF and exercise enhance neuronal DNA repair by stimulating CREB-mediated production of apurinic/apyrimidinic endonuclease 1. Neuromol Med.

[b111] 111Autry AE, Bambah-Mukku D. The role of brain-derived neurotrophic factor in neural circuit development and function[M]//Rubenstein J, Rakic P, Chen B, et al. Synapse Development and Maturation: Comprehensive Developmental Neuroscience. 2nd ed. London: Academic Press, 2020: 443–466.

[112] (2012). Role of neurotrophins in the development and function of neural circuits that regulate energy homeostasis. J Mol Neurosci.

[113] (2017). Effects of gravity changes on gene expression of BDNF and serotonin receptors in the mouse brain. PLoS One.

[114] (2016). The "proactive" model of learning: integrative framework for model-free and model-based reinforcement learning utilizing the associative learning-based proactive brain concept. Behav Neurosci.

[115] (2022). Function of brain-derived neurotrophic factor in the hypothalamus: implications for depression pathology. Front Mol Neurosci.

[116] (1998). Expression of brain-derived neurotrophic factor and its cognate receptor, TrkB, in the rat suprachiasmatic nucleus. Exp Neurol.

[117] (2011). Aerobic exercise improves hippocampal function and increases BDNF in the serum of young adult males. Physiol Behav.

[118] (2011). Running is the neurogenic and neurotrophic stimulus in environmental enrichment. Learn Mem.

[119] (2004). Hippocampal BDNF mediates the efficacy of exercise on synaptic plasticity and cognition. Eur J Neurosci.

[120] (2016). FNDC5/irisin, a molecular target for boosting reward-related learning and motivation. Med Hypotheses.

[121] (1998). Circadian rhythm of brain-derived neurotrophic factor in the rat suprachiasmatic nucleus. Neurosci Lett.

[122] (2000). Role of brain-derived neurotrophic factor in the circadian regulation of the suprachiasmatic pacemaker by light. J Neurosci.

[123] (2005). Overlap in the distribution of TrkB immunoreactivity and retinohypothalamic tract innervation of the rat suprachiasmatic nucleus. Neurosci Lett.

[124] (2006). Brain-derived neurotrophic factor and neurotrophin receptors modulate glutamate-induced phase shifts of the suprachiasmatic nucleus. Eur J Neurosci.

[125] (2006). Constitutive activation of ras in neurons: implications for the regulation of the mammalian circadian clock. Chronobiol Int.

[126] (2017). Ras activity tunes the period and modulates the entrainment of the suprachiasmatic clock. Front Neurol.

[127] (2013). Brain-derived neurotrophic factor/TrkB signaling regulates daily astroglial plasticity in the suprachiasmatic nucleus: electron-microscopic evidence in mouse. Glia.

[128] (2006). Brain-derived neurotrophic factor regulation of N-methyl-D-aspartate receptor-mediated synaptic currents in suprachiasmatic nucleus neurons. J Neurosci Res.

[129] (1998). BDNF acutely increases tyrosine phosphorylation of the NMDA receptor subunit 2B in cortical and hippocampal postsynaptic densities. Mol Brain Res.

[130] (1997). Brain-derived neurotrophic factor and nerve growth factor potentiate excitatory synaptic transmission in the rat visual cortex. J Physiol.

[131] (2013). Molecular and neural bases underlying roles of BDNF in the control of body weight. Front Neurosci.

[132] (2016). Stressed neurons protect themselves by a tissue-type plasminogen activator-mediated EGFR-dependent mechanism. Cell Death Differ.

[133] (2018). Neuron activity–induced Wnt signaling up-regulates expression of brain-derived neurotrophic factor in the pain neural circuit. J Biol Chem.

[134] (1998). CREB in the mouse SCN: a molecular interface coding the phase-adjusting stimuli light, glutamate, PACAP, and melatonin for clockwork access. J Neurosci.

[135] (2002). Phosphorylation of CREB Ser142 regulates light-induced phase shifts of the circadian clock. Neuron.

[136] (2007). An inhibitor of casein kinase Iϵ induces phase delays in circadian rhythms under free-running and entrained conditions. J Pharmacol Exp Ther.

[137] (2007). Protein kinase C modulates the phase-delaying effects of light in the mammalian circadian clock. Eur J Neurosci.

[138] (2013). Protein kinase C differentially regulates entrainment of the mammalian circadian clock. Chronobiol Int.

